# An effective strategy for development of docetaxel encapsulated gold nanoformulations for treatment of prostate cancer

**DOI:** 10.1038/s41598-020-80529-1

**Published:** 2021-02-02

**Authors:** S. Thambiraj, R. Vijayalakshmi, D. Ravi Shankaran

**Affiliations:** 1 Nano-Bio Materials and Sensors Laboratory, National Centre for Nanoscience and Nanotechnology, University of Madras, Guindy Campus, Chennai, Tamil Nadu 600 025 India; 2 Department of Preventive Oncology, Cancer Institute (WIA), Adyar, Chennai, 600 020 India

**Keywords:** Materials science, Nanoscience and technology

## Abstract

Nanoformulation based drug delivery is one of the most important research areas in the field of nanomedicine, which provides promising alternatives to the limitations of conventional chemotherapy. Nano drug delivery enables improved pharmacokinetic profile, bioavailability and therapeutic efficiency compared to the regular chemotherapeutic drugs. Herein, we have established a simple method for the synthesis of docetaxel (Dtx) encapsulated poly (ethylene glycol) (PEG) functionalized gold nanoparticles (AuNPs) for targeted drug delivery to prostate cancer. AuNPs were synthesized by the citrate ion reduction method followed by functionalization with thiol-PEG-amine (SH-PEG-NH_2_). SH-PEG-NH_2_ functionalized AuNPs were conjugated with the targeting vehicle, folic acid (FA). The anticancer drug, Dtx was encapsulated within AuNPs by the non-covalent linkage method. The physicochemical characteristics of the synthesized nanoformulations were extensively characterized by various spectral and microscopic studies. HR-TEM indicates the average size of the AuNPs is 16 nm and the nanoformulations is 18 nm. The encapsulation efficiency of the Dtx is ~ 96% which is confirmed by the elemental mapping analysis. The in vitro drug release profile of Dtx and AuNPs nanoformulations were studied by the dialysis membrane method. The anticancer activity of docetaxel encapsulated AuNPs were evaluated with prostate cancer cell lines (PC3). The drug encapsulated nanoformulations reduced the cell viability to about 40% (40 µM concentration at 24, 48 and 72 h of treatment). The optical microscopy observation reveals that the damage of prostate cancer cells after exposure to Dtx encapsulated AuNPs. The good cytotoxic activity of the present nanoformulation against prostate cancer cell lines enables its application for targeted drug delivery to prostate cancer.

## Introduction

Nanomedicine is an important research area which plays a significant role in the health and wealth of current and future generation. Nanomedicine involves the design and fabrication of highly desirable and tailor-made nanoformulation for efficient use in diagnosis and drug delivery. The nanoformulations have unique salient features to transport and deliver the drug to the various biological entities for diagnosis, imaging and treatment of various diseases^1–5^. In the biological transport process, the actions of nanoformulations are challenged by the internal and external barriers (skin, mucosa, blood, extracellular matrix and cellular membranes) and various physical characteristics such as size, shape, surface charges and intrinsic chemical properties^6–10^. Due to their unique structural properties including the large surface area and long blood circulation time, nanoparticles are promising candidate for optimizing therapy to any disease^11,12^. In fact, the size, shape and structural characteristics of nanoparticles play a vital role in the biodistribution of in vivo analysis^13^.

Over the years, the advancements in nanotechnology, materials science and biochemical processes resulted in the development of advanced nanoformulations with desired functionalities for specific applications. The biocompatible nanocarriers like liposome, dendrimers, polymeric nanoparticles, and inorganic nanoparticles have been used for more efficient and safer delivery of a myriad of drugs exploring the advantages of long blood circulation time, improved pharmacokinetics and reduced the side effects^2,14–16^.

Docetaxel (Dtx) is a semisynthetic anticancer mitotic ("antineoplastic" or "cytotoxic") chemotherapy drug in the *taxoid* family. It is derived from the European yew tree (*Texus baccata*). The Dtx is recommended for optional treatment in cancer patients with hormone-refractory metastatic prostate cancer^17–21^. Dtx is a chemotherapeutic medication (antitumor activity) against a wide range of solid tumors, including breast, lung, head, prostate, neck, non-small cell lung and ovarian cancers^19–26^. Many researchers have been established that Dtx bind to β-tubulin, which interferes with the normal function of the microtubules polymerization dynamics, dividing cell mitosis, interface microtubule function and triggering apoptosis^27,28^. Dtx has the limitations of low water solubility, severe allergic reactions and systemic toxicity^29^. To overcome these drawbacks of Dtx in clinical use, nanoformulations based drug delivery systems like liposome^30^, inorganic nanoparticles^25^, magnetic nanoparticles^31^, polymeric nanoconjugates^32^ and nanotubes^33^ that have been formulated for Dtx delivery.

Gold based nanoformulations are promising vehicles for Dtx administration due to fascinating properties including tunable size and shape, biocompatibility and facile conjugation to biomolecules^34–37^. Moreover, the unique optical, electrical, physical and chemical properties (size and shape) of gold nanoparticles (AuNPs) make them an excellent candidate for biomedical applications^38^, including targeted drug delivery^39^, photothermal therapy^40^, cancer diagnosis^41^ and tumor imaging^42^. The surface plasmon resonance (SPR) properties of the AuNPs play a major role in the biological system to allow their characterization and detection properties^43^.

Various synthesis methods have been used for the synthesis of AuNPs and nanoformulations. Surface functionalization is one of the most important option for the development of nanoformulations with enhanced recognition and biocompatibility for biomedical applications^44^. It has been reported that functionalization of nanoformulations with folic acid (FA) enables targeted delivery of drugs due to its interaction with folate receptors surrounding the cancer cells^45^. Ngernyuang et al. reported the conjugation of FA onto the surface of PEG functionalized AuNPs by simple physical agitation and mixing method^46^. Neshastehriz et al. demonstrated FA conjugation with AuNPs by mixing of FA onto the citrate-stabilized AuNPs by ultra-probe-sonication^47^. However, the stability of the FA conjugated AuNPs has to be increased for its prolonged and safe action under physiological conditions (pH and temperature). Hence, interest has been shown to formulate the covalent attachment of FA onto the surfaces of the AuNPs. Owing to the high affinity of the thiol (SH) groups toward AuNPs, bi-functional (SH-PEG-NH_2_) molecules can be used for functionalization enabling the formation of the gold-sulfur bond (Au-S-PEG-NH_2_) and free functional groups of alkyne, carboxylate, and amine groups may be used for covalent coupling with FA. This conjugation process has to be carefully optimized for better performance of the nanocarrier^48–51^.

Cancer (an abnormal and uncontrolled proliferation of cells) is a serious disease leading to increasing mortality throughout the world. According to the National Cancer Institute (NCI), around 14 million new cases were admitted and 26 million cancer-related deaths from 1991 to 2016. About 40% of cancer patients may be treated and diagnosed during their lifetime. Currently, treatment and diagnosis of cancer are a high demand that remains an ongoing challenge in oncology (pathophysiological and heterogeneous disease)^52,53^. Prostate cancer (PCa) is the second most common disease in men and which leading causes of death in men^54^. In a 2019 survey of PCa, around 1,64,690 new cases and 29,430 deaths caused in the United States. However, PCa was typically treated by radical prostatectomy (surgery), hormonal therapy, chemotherapy and radiation therapy. While the treatment, cancer patients affect some side effects in different ways such as infertility, incontinence urinary, reduced sexual desire and hormone-based side effects^53,54^. In these aspects, various research groups are focused on developing effective imaging and drug delivery methods for prostate cancer. In general, cancer chemotherapy causes severe toxicity due to the indiscriminate distribution of anticancer drugs upon systemic administration^54^. Therefore, selective delivery of these therapeutic drugs to a target tumor cell is desirable. Different cell-specific markers/receptors are overexpressed in various cancer cells which can be exploited for enhanced cancer selectivity and endocytosis. The overexpressed FA binding receptors on prostate cancer will be targeted here by functionalizing the nanocarrier with respective ligands^55^. The current treatment options available for cancer therapies are inadequate and spur demand for improved technologies. Rapid growth in nanotechnology towards the development of nanomedicine provides a wide range of new materials, tools and possibilities, from earlier diagnostics and improved imaging and therapies.

In the present work, we have established the sequential process for the synthesis of gold nanoformulations (AuNPs-PEG-FA-Dtx): (i) AuNPs was synthesized by citrate ion (reducing and stabilizing agent) reduction method, (ii) AuNPs were functionalized with SH-PEG-NH_2_ via the formation of an Au−S bond by the addition method, (iii) the targeting ligand of FA was conjugated with the PEG functionalized AuNPs via *N*-ethyl-*N*-(3-dimethylamino propyl) carbodiimide/*N*-hydroxy succinamide (EDC/NHS) by the coupling method followed by covalent linkage method and (iv) the anti-cancer drug, Dtx were encapsulated with AuNPs-PEG-FA by the non-covalent linkage method. The spectral, diffraction features and microscopic characteristics of the synthesized AuNPs-PEG-FA-Dtx were examined by various analytical techniques. The encapsulation efficiency and drug release profile of the nanoformulations were examined. The anticancer activity and drug delivery efficacies of AuNPs nanoformulations, against prostate cancer cell line (PC3) was investigated in both free drug and drug encapsulated nanoformulations.

## Experimental section

### Materials

Gold (III) chloride trihydrate (HAuCl_4_.3H_2_O, M.W. 393.83 g/mol), docetaxel (Dtx) (C_43_H_53_NO_14_, M.W. 807.879 g/mol), folic acid (FA) (C_19_H_19_N_7_O_6_, MW. 441.40 g/mol), *N*-ethyl-*N*-(3-dimethyl aminopropyl) carbodiimide (EDC) (C_8_H_17_N_3_, MW.155.245 g/mol), *N*-hydroxy succinimide (NHS) (C_6_H_5_NO_3_, MW.115.09 g/mol), Trypsin EDTA, Ham’s F12 (F-12 nutrient medium), l-Glutamine (C_5_H_10_N_2_O_3_, MW.146.146 g/mol), sodium bicarbonate (NaHCO_3_), non-essential amino acids and fetal bovine serum (FBS) were procured from Sigma Aldrich Chemicals, USA. Trisodium citrate (Na_3_C_6_H_5_O_7_, MW. 258.06 g/mol), *N*,*N*-dimethyl sulfoxide (DMSO) (C_2_H_6_OS), disodium hydrogen phosphate (Na_2_HPO_4_, MW.141.96 g/mol) and orthophosphoric acid (H_3_PO_4_, MW.97.994 g/mol) were purchased from Merck Chemicals, Mumbai. Ethanol (99.9%) was received from Changshu Hongsheng Fine Chemical Co., Ltd, China. Premix WST-1cell proliferation assay kit was procured from Takara scientific company (USA). Human prostate cancer cell lines of PC3 were received from National Centre for Cell Science (NCCS), Pune, India. PC3 cells were maintained with Ham’s F12 (F-12 Nutrient Medium) and 10% FBS (maintenance medium). All the chemicals were used without any purification and Millipore water was used throughout the experiment.

## Methods

### Synthesis of gold nanoparticles

The modified procedure has been used for the synthesis of gold nanoparticles from gold (III) chloride trihydrate by chemical reduction method using trisodium citrate as a reducing agent^25,56–58^.

**Figure 1:**
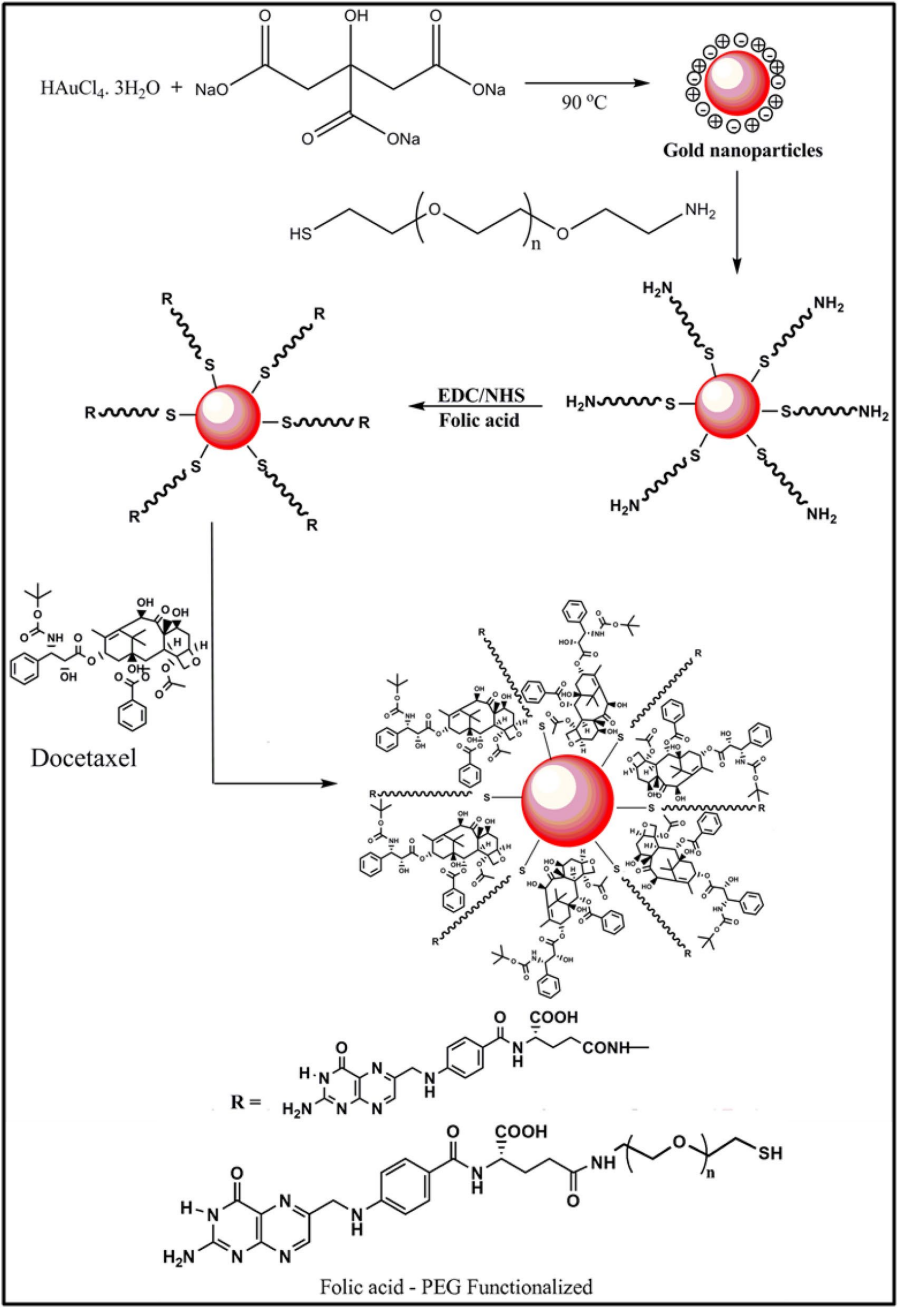
Mechanism for the synthesis of gold nanoformulations.

Briefly, 5 mL of 1 wt% of trisodium citrate in aqueous medium was added by dropwise to 200 mL aliquot of 1 mM HAuCl_4_·3H_2_O boiling with stirring the solution under reflux condition. After adding the reducing agent, the reaction mixture color was changed from golden yellow to colorless. This reaction was continued until the solution turned to wine red. This color change appeared within 3 min due to the reduction of Au ^(III)^ to Au^0^ and the reaction was completed within 5 min. The obtained colloidal suspension was allowed to cool at room temperature and the colloidal suspension was sonicated for 15 min at 37 kHz. Followed by, the loosely bounded citrate ions were removed by centrifugation for 30 min at 0 °C (10,000 rpm). The resulted AuNPs solution was stored at 4 °C and used further characterization. Figure 1 illustrates the mechanism for the synthesis of gold nanoformulations.

### Functionalization of gold nanoparticles

Synthesized citrate capped AuNPs were functionalized with SH-PEG-NH_2_. 2 mg of SH-PEG-NH_2_ was dissolved in 5 mL Millipore water, then added drop-wise into 100 mL of 1 mM concentration of AuNPs solution and stirred for 12 h at room temperature. After functionalization, the solution was maintained the pH range of 6.5 to prolong the stability of the nanoparticles. Further, the solution was sonicated for 30 min and centrifuged against Millipore water at 10,000 rpm for 30 min at 0 °C. The stable SH-PEG-NH_2_ functionalized AuNPs solution was stored at 4 °C and used further characterization.

### Fabrication of FA conjugated AuNPs

In order to fabricate FA conjugated AuNPs, 1 mM of FA was dissolved in 10 mL of DMSO and sonicated for 15 min. The sonicated solution was centrifuged at 10,000 rpm for 20 min at 0 °C. The prepared FA solution was mixed with *N*-(3-dimethyl aminopropyl)-*N′*-ethylcarbodiimide hydrochloride (EDC) and *N*-hydroxy succinamide (NHS) and the molar ratio of the FA: EDC: NHS was of 10:1:1 and the reaction solution was maintained the pH at 6.5. The reaction mixture was continuously stirring for 12 h followed by sonication and centrifuged at 10,000 rpm for 30 min. The filtered solution was mixed with SH-PEG-NH_2_ functionalized AuNPs and stirred continuously for 6 h. The resulted solution was sonicated and centrifuged at 10,000 rpm for 10 min at 0 °C for three times. The fabricated AuNPs/PEG/FA solution was stored at 4 °C and used further characterization.

### Fabrication of docetaxel encapsulated AuNPs

1 mM of Dtx was dissolved in 5 mL ethanol, water and tween 80 (1:1:0.25 ratio) solution and sonicated for 15 min. The sonicated solution was centrifuged at 10,000 rpm for 20 min at 0 °C. This solution was added into the SH-PEG-NH_2_ functionalized AuNPs in 50 mL beaker using the non-covalent binding method with continuously stirred for 4 h. The prepared AuNPs/PEG/Dtx/FA solution was sonicated in an ice bath for 30 min, followed by centrifuging at 10,000 rpm for 20 min at 0 °C. The obtained solution was stored at 4 °C and used for further characterization.

### Characterization of Dtx encapsulated gold nanoformulations

The physicochemical properties and step-by-step conjugation of the synthesized AuNPs nanoformulations were extensively characterized by various analytical techniques. UV–Vis absorption spectra were carried out by using Agilent diodaris spectrophotometer and Cary-8453, USA. The surface functional groups of the nanoformulations were determined by FT-IR spectrophotometer (Bruker, Vertex, 80 V, USA). The crystallinity and phase purity of the nanoformulations were studied by XRD analysis (Bruker D8 advance PXRD and Rigaku X-ray diffractometer, Smart lab, UK). The chemical composition and oxidation state were evaluated by X-ray photoelectron spectroscopy (XPS, Omicron Nanotechnology, ESCA-14 (Germany). The surface morphology and elemental composition of the nanoformulations were examined by a high-resolution transmission electron microscope (HR-TEM) ((JEOL-2100-JEM) and (Bruker)) and field emission scanning electron microscope (FE-SEM) (Hitachi, SU-6600—Japan) instrument with energy dispersive X-ray spectroscopy (EDS). The chemical bond and surface modification of the nanoformulations were determined by Raman spectrophotometer (Xplora Plus, Raman spectrometer, Horiba, Japan with laser excitation of 785 nm). The anticancer activity of the AuNPs nanoformulations was analyzed by ELIZA Reader (Enzyme-Linked Immunosorbent Assay) microplate reader (Robonik, Mumbai, India) with the excitation wavelength of 460 nm.

### Encapsulation efficiency of gold nanoformulations

The percentage of drug encapsulated in gold nanoformulations was determined by separating the un-entrapped drug from nanoformulations by centrifugation at 10,000 rpm for 30 min using cooling centrifuge (CPR-Plus 24, Remi Instruments, Mumbai, India). The clear supernatant was analyzed for the contents of Dtx by measuring absorbance in a UV–Visible spectrophotometer at λmax = 230 nm^59^. The percentage encapsulation efficiency and loading capacity were calculated by the following equation,

$$ {\text{Encapsulation }}\;{\text{efficiency}} = {\text{Amount }}\;{\text{of}}\;{\text{ the }}\;{\text{drug }}\;{\text{in }}\;{\text{formulation}}/{\text{Total }}\;{\text{amount}}\;{\text{ of }}\;{\text{drug }} \times { 1}00 $$

where Dtx_t_ is the total amount of Dtx used in the preparation of nanoformulations and Dtx_f_ is the unentrapped Dtx present in the supernatant.

### In vitro drug release

In vitro drug release kinetics of optimized Dtx encapsulated AuNPs nanoformulations and the free drug was investigated by the dialysis membrane method. Briefly, 75 mL of phosphate-buffered solution (pH 7.4) and 5 mL of 0.1% tween 80 was taken as release media in 150 mL borosil glass beaker. After that, 5 mL of nanoformulations were poured into the dialysis membrane (molecular weight cut-off 12–14 kDa) and immersed in an 80 mL PBS solution with the help of a glass rod. The bag was immersed centrally into the release media using a burette stand. The beaker was placed on the magnetic stirrer with maintained a constant rotation of 300 rpm with a magnetic pellet. The release kinetics was predetermined at different time intervals like 1–72 h. At a notable time interval, 3 mL of drug release medium was aspirated and replaced 3 mL fresh drug release media and also maintained the constant bath volume. The absorbance of the samples was recorded in a UV–Vis spectrophotometer at λmax of Dtx at 230 nm, FA at 280 and 344 nm, and AuNPs at 528 nm. The calibration curve was calculated with different concentrations of Dtx in the PBS solution.

The in vitro drug release kinetics were analyzed by different release kinetic models like zero-order, first-order, Higuchi kinetics, Korsmeyer–Peppas model, and Hixon–Crowell model which enables the identification of the R^2^ value (correlation coefficient)^60^.

### Cytotoxicity analysis of gold nanoformulations

In vitro cytotoxicity analysis used to investigate the cell viability or metabolic activity of prostate cancer cell line (PC3) against gold nanoformulations. This colorimetric assay is highly sensitive and reliable to the transformation of the metabolic activity of the cells. Briefly, 100 µL (2500 cells per well) of PC3 cells were harvested, counted, and seeded in flat-bottom 96 well plate (Corning USA) and then incubated for 24 h at 37 °C under 5% CO_2_ atmospheric conditions which used to adhere the cells. After 24 h, the cells were washed with PBS (Phosphate Buffer Saline solution) solution twice. Subsequently, the cells were treated with different concentration of synthesized AuNPs nanoformulations (10 to 60 µM) includes free AuNPs (10 to 125 µM), PEG functionalized AuNPs (10 to 125 µM), FA (10 to 60 µM), and Dtx (10 to 60 µM) and incubated for different time intervals like 24 h, 48 h, and 72 h. The AuNPs nanoformulations free cells are considered as control and the experiments were carried out in triplicate. The selected concentration of the nanoformulations which enables the drug particles were reached in the cancerous cells at the plasma level. After the incubation time interval, the HAMS F12 medium was aspirated and washed with PBS two times. Then, 3 µL of Premix WST assay solution was added in each well and the cells incubated for 4 h in dark condition. In this period, the metabolically active cells to transform WST into the formation of insoluble Formazan crystals. Followed by, the optical absorbance of cells was measure immediately at 460 nm using ELIZA (Enzyme-Linked Immunosorbent assay) microplate reader (Robonik, Mumbai, India). All the experiments were carried out in triplicate. The cell viability was calculated by the following equations.

$$ {\text{Cell viability }}\left( \% \right) \, = \, \left( {{\text{Abs}}_{{{\text{sample}}}} /{\text{Abs}}_{{{\text{control}}}} } \right) \, \times { 1}00 $$

### Statistical analysis

The statistical analysis was expressed as a mean ± standard deviation by SPSS 16.0 software (Name: IBM SPSS Software, Version Number: SPSS16.0, and URL Link: https://www.ibm.com/in-en/products/spss-statistics.Chicago, IL,USA).The statistical comparison was carried out all the AuNPs nanoformulations by one-way analysis of variance (ANOVA) by post hoc hypothesis testing (Tukey test). The p-value is less than 0.05 (p < 0.05) was considered to be a significance level.

## Results and discussion

### Synthesis of Dtx encapsulated AuNPs nanoformulations

**Figure 2:**
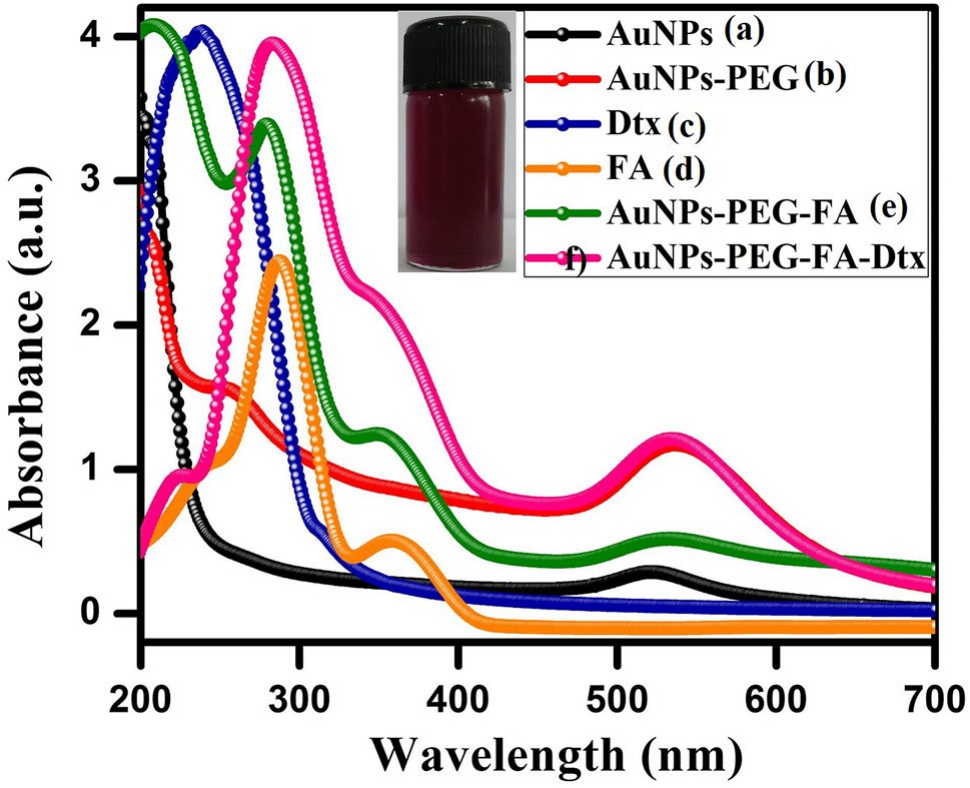
UV–Vis absorption spectra of the synthesized gold nanoformulations (**a**) AuNPs (λmax = 527 nm), (**b**) AuNPs-SH-PEG-NH_2_ (λmax = 528 nm), (**c**) Dtx (λmax = 231 nm), (**d**) FA (λmax = 277 and 344 nm), (**e**) AuNPs-PEG-FA (λmax = 277, 344 and 528 nm) and (**f**) AuNPs nanoformulations (λmax = 228, 277, 344 and 528 nm) and inset photographic image of gold nanoformulations at pH = 6.5.

The stability of the colloidal AuNPs is a very important feature for surface functionalization. The colloidal suspension of AuNPs was synthesized by chemical reduction method (citrate ion acts as a reducing agent and stabilizing agent). UV–Vis absorption spectra of the citrate capped AuNPs exhibited the Surface Plasmon Resonance (SPR) band at 527 nm as shown in Fig. 2a. The magnitude of the SPR peak depends on the concentration and the morphology of the synthesized AuNPs. This absorption spectrum indicates that the increased concentration of the AuNPs and the SPR peak height also increased to some extent the absorption peak gets broaden. Followed by, AuNPs were functionalized with thiolated PEG amine (SH-PEG-NH_2_). During the thiolation of AuNPs, AuNPs appeared rapid agglomeration which exhibited the SPR signal of PEG functionalized AuNPs observed at 528 nm (Fig. 2b). To overcome this agglomeration of AuNPs, during the thiolation process the AuNPs solution pH was adjusted at 6.5^61^. This pH condition, PEG functionalized AuNPs are highly stable to prevent the agglomeration^62^. The stable solution of PEG functionalized AuNPs were conjugated with Au–S bond and amine (–NH_2_) groups. Thereafter, PEG functionalized AuNPs were conjugated with FA by using EDC/NHS coupling method (the free amine (–NH_2_) groups were bonded with the carboxylic group (–COOH) and formed a strong bond between PEG-AuNPs and FA). UV–Vis absorption spectra of FA were observed in the two bands at 277 and 367 nm (Fig. 2d). The FA conjugated PEG functionalized AuNPs, it was observed that the one molecule of FA was attached per six molecules of PEG functionalized AuNPs. This result indicates that the FA was partially attached over the surface of PEG functionalized AuNPs, which exhibited the steric hindrance of the bulky structure of FA. The SPR signal of the FA conjugated PEG functionalized AuNPs indicated that the 528 nm, 344 nm, and 277 nm (Fig. 2e). These results suggest that the FA were strongly attached over the surfaces of PEG functionalized AuNPs. Subsequently, the anticancer drug of Dtx was conjugated by the non-covalent linkages method, which enables the physical adsorption of Dtx with FA conjugated PEG functionalized AuNPs. UV–Vis spectra of the Dtx were observed at 231 nm ascribed to π–π* transition of carbonyl and hydroxyl groups were present in the sample (Fig. 2c). During the synthesis of AuNPs nanoformulations (PEG-AuNPs-FA-Dtx), various reaction parameters were optimized such as the concentration of the reactant, concentration of reducing agent, temperature, reaction time, and pH, which enables the formation of highly stable nanoformulations. The resulted AuNPs nanoformulations were examined by UV–Vis absorption spectroscopy. AuNPs nanoformulations (AuNPs-PEG-FA-Dtx) were observed at the peak at 228, 279, 355, and 528 nm (Fig. 2f). These results indicate the formation of AuNPs nanoformulations with step-by-step conjugation of FA and Dtx.

### Characterization studies of nanoformulations

#### Surface structural analysis

**Figure 3:**
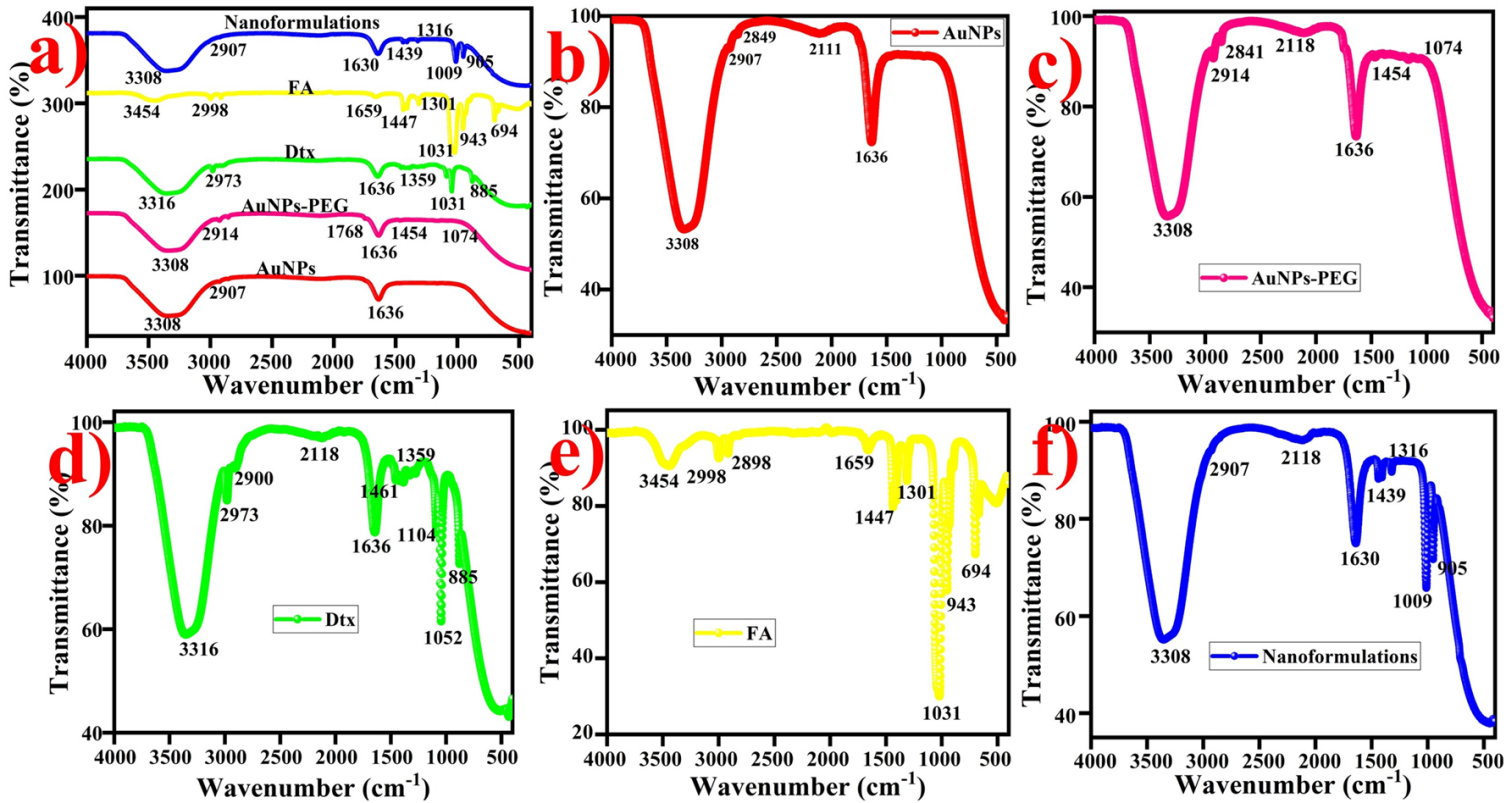
FTIR spectra of gold nanoformulations, (**a**) combined spectra of nanoformulations, (**b**) AuNPs, (**c**) AuNPs-PEG (**d**) Dtx, (**e**) FA and (**f**) AuNPs nanoformulations (AuNPs-PEG-FA-Dtx).

The surface functional groups and chemical bonding of the synthesized nanoformulations were examined by FTIR spectra shown in Fig. 3a. The FTIR spectrum of citrate capped AuNPs exhibits shoulder band at 3301 cm^−1^ and 1636 cm^−1^ ascribed to the O–H stretching vibration and C=C stretching vibration of carbonyl groups is present in the AuNPs, respectively (Fig. 3b). The broad band at 2111 cm^−1^ corresponds to the CO_2_ (atmosphere)/CΞC week stretching vibration of alkyne. The doublet peak appeared at 2907 cm^−1^ and 2849 cm^−1^ attributed to the C–H stretching vibration of carboxyl groups also present in the AuNPs. PEG functionalized AuNPs exhibited the major signals at 3305 cm^−1^, 2906 cm^−1^ and 2841 cm^−1^ (O–H and C–H stretching vibration of carbonyl groups), 2118 cm^−1^ (atmospheric CO_2_/CΞC week stretching vibration of alkyne), 1765 cm^−1^ (C=O strong stretching vibration), 1636 cm^−1^ (C=C stretching vibration of carboxylic acid) 1454 cm^−1^ (S=O stretching vibration of sulfonate) and 1074 cm^−1^ (C–N stretching vibration of amine) present in the sample (Fig. 3c)^62^. FTIR spectrum of the Dtx indicates the sharper band at 3316 cm^−1^, 2973 cm^−1^ and 2885 cm^−1^ attributed to the N–H and C–H stretching vibration of secondary amine and alkane, respectively (Fig. 3d)^64^. A small intense peak at 1636 cm^−1^ corresponds to the N–H bending vibration, 1447 cm^−1^ ascribed to the C–H bending vibration of CH_2_ and CH_3_ and a week band at 1359 m^−1^ corresponds to the O–H bending vibration of carboxylic group. A band at 1104 cm^−1^, 1057 cm^−1^ and 885 cm^−1^ corresponds to the C=O, C–N and C=C bending vibration of carbonyl, amide and alkane groups are appeared in the Dtx, respectively. The appearance of the FTIR spectrum of FA exhibited the characteristic peaks at 3454 cm^−1^ (primary amine), 2998 cm^−1^ and 2898 cm^−1^ (C–H stretching vibration of alkane), and 1659 cm^−1^ (C=N amine, conjugated double bond). The doublet band at 1447 cm^−1^ and 1301 cm^−1^ representing C–H bending vibration and bending of alkene are present in the FA, respectively^63^. The peaks at 1031 cm^−1^ and 943 cm^−1^ (S=O/C–N and C=C stretching vibration of sulfoxide/amide and alkane) present in the FA (Fig. 3e). It is evident that the peak at 3454 cm^−1^, 1659 cm^−1^ and 1031 cm^−1^ corresponds to the formation of primary amine and sulfoxide groups are conjugated over folic acid using EDC/NHS coupling method.

FTIR spectra of AuNPs nanoformulations exhibits the major signals at 3308 cm^−1^ corresponds to the OH stretching vibration of carboxylic groups (Fig. 3f). The band at 2907 cm^−1^ corresponds to the CH stretching vibration of alkanes. The band at 2118 cm^−1^ corresponds to the atmospheric CO_2_/CΞC week stretching vibration of alkyne. The strongest peak at 1630 cm^−1^ attributed to the C=C stretching vibration of alkane. The intense band at 1439 cm^−1^ and 1316 cm^−1^ ascribed to the C–H and O–H plane bending vibration of alkanes. The weak band observed at 1009 cm^−1^ is attributed to the C–N/S=O stretching vibration of amine and sulfonate are present in the nanoformulations^65^. A small band at 905 corresponds to the C=C bending vibration of alkane is present in the nanoformulations. Evidently, the FTIR spectra exhibited the FA conjugation with PEG functionalized AuNPs via amide bond as well as the attachment of Dtx onto the AuNPs-PEG-FA-Dtx nanoformulations via hydrogen bonding.

#### The crystalline structure of gold nanoformulations

**Figure 4:**
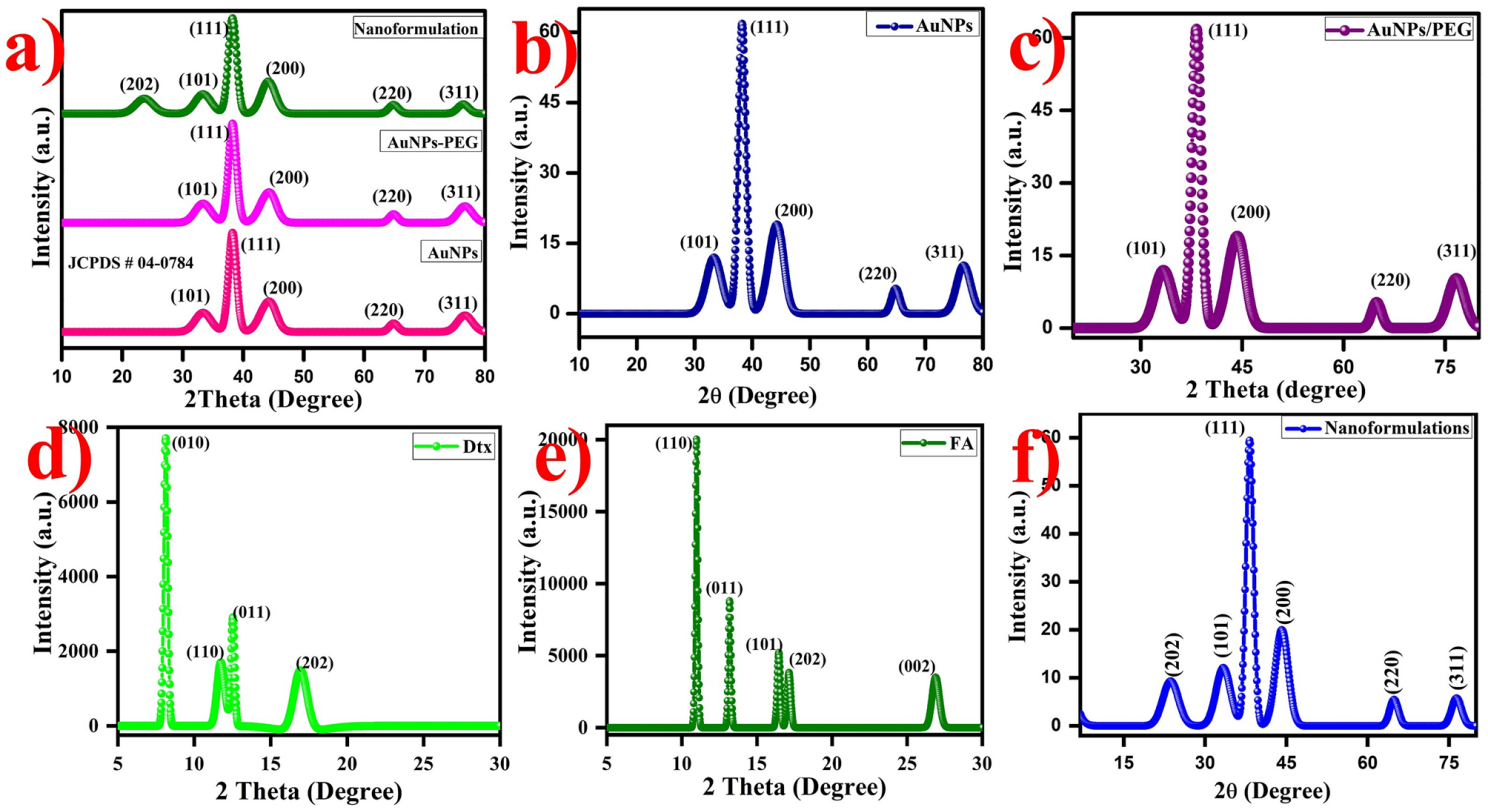
XRD pattern of the gold nanoformulations (**a**) Combined spectra of AuNPs nanoformulations, (**b**) AuNPs (**c**) AuNPs-PEG, (**d**) Dtx (**e**) FA and (**f**) gold nanoformulations were observed the FCC crystal structure.

The crystallinity, phase purity, stability and chemical bonding of the AuNPs nanoformulations were determined by XRD analysis as shown in Fig. 4a. XRD pattern exhibits the diffraction peaks at 2θ = 36.45°, 38.21°, 44.39°, 64.76° and 77.76° corresponds to the Bragg reflection signals at (101), (111), (200), (220) and (311) indicates the standard metallic gold (Au^0^) [(Joint Committee on Powder Diffraction Standards, USA-JCPS ID # 00-002-1095] with lattice parameters of a = 4.065 Å and the space group of Fm-3 m. This Bragg reflection signals exhibited the face centered cubic (FCC) crystal structure and the shoulder peak was observed around 30–40° which attributed to the highly crystalline nature of AuNPs (Fig. 4b)^66,67^. The size of the nanoparticles was measured indirectly using a broad bottom width of (111) reflection which indicates the smaller size of the nanoparticles. Debye–Scherrer’s equation was used to determine the size of the nanoparticles by (111) width of the Bragg reflection signal. The size of the AuNPs was found to be ~ 18 nm. The (111) reflection peak exhibited the strong shoulder peak rest of the peaks are weak peaks which indicate the predominant orientation. There is no small impurities peaks were observed which shows the synthesized AuNPs are highly pure. Interestingly, the XRD pattern of the SH-PEG-NH_2_ functionalized AuNPs exhibited diffraction band is similar to AuNPs (Fig. 4c), whereas, the XRD pattern of the characteristics diffraction peak of FA at 10.9°, 13.0°, 16.2°, 16.90°, and 27.2° corresponds to the (110), (011), (101), (220) and (002), respectively (Fig. 4d)^68^. The XRD pattern of the pure Dtx exhibits the signals at 2θ values of 8.2°,12.18°, 14.12°, 16.89° corresponds to the (010), (110), (011) and (202) crystal planes, respectively (Fig. 4e)^69^.

Finally, the XRD pattern of the synthesized AuNPs-PEG-FA-Dtx exhibits the diffraction signals at 2θ values of 22.39°, 36.45°, 38.21°, 44.39°, 64.76° and 77.76° corresponds to the (202), (101), (111), (200), (220) and (311) crystal planes, respectively (Fig. 4f). On comparison with FA, Dtx and AuNPs, the diffraction peaks of the FA and Dtx signals are slightly suppressed due to the strong attachment between FA-AuNPs-PEG-Dtx. Thus, the XRD analysis confirmed the obtained AuNPs were FCC crystal structure and the step-by-step functionalization of SH-PEG-NH_2_ and FA over AuNPs and Dtx encapsulated over the surface of AuNPs-PEG-FA. The synthesized AuNPs nanoformulations were highly stable in the aqueous medium over the period of one year at room temperature.

#### Morphological and elemental mapping analysis of AuNPs nanoformulations

**Figure 5:**
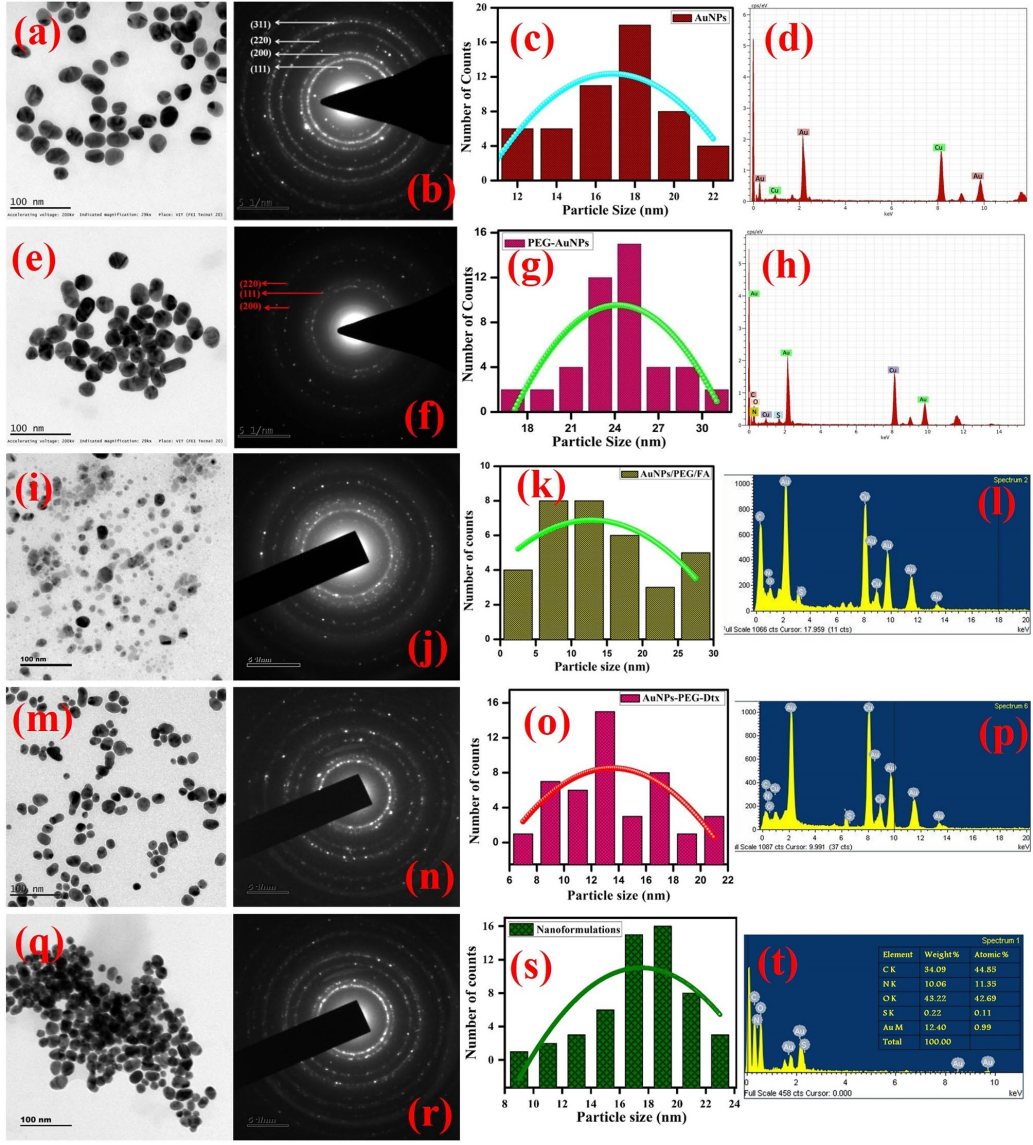
HR-TEM images of the gold nanoformulations (**a**–**d**) AuNPs TEM image, SAED, particle size distribution and EDS spectrum followed by the similar characteristics were carried for remaining particle, (**e**–**h**) PEG functionalized AuNPs, (**i**–**l**) FA conjugated AuNPs-PEG, (**m**–**p**) Dtx loaded AuNPs-PEG and (**q**–**t**) drug encapsulated gold nanoformulations.

The surface morphology and microstructure of the synthesized gold nanoformulations were determined by HR-TEM analysis. Typical TEM micrographs of the citrate capped AuNPs indicates that the particles are spherical in shape and uniformly distributed over the copper substrate with an average dimension of 16 nm (Fig. 5a–d). AuNPs were uniformly attached over the SH-PEG-NH_2_ (Fig. 5e–h). The average diameter is 24 nm, the slight increase in the dimension of AuNPs is due to the strong interaction between (Au–S) AuNPs-PEG. Figure S1a–d exhibits the FA particles are spherical in shape and the particles are observed the average dimension of 90 nm. After, FA conjugate over the surface of AuNPs-PEG exhibited particles are spherical in shape with an average dimension of 16 nm with slight changes in their shape (Fig. 5i–l). This result confirms that the FA conjugated over the AuNPs-PEG and the interaction between FA and AuNPs through hydrogen bonding. Figure S1e–h depicts the Dtx particles observed the core–shell morphology and uniformly distributed over the substrate with an average dimension of 290 nm. Figure 5m–p exhibits the Dtx encapsulated PEG functionalized AuNPs observed the particles are spherical in shape with slight changes in the size of the particles. Finally, the gold nanoformulations (AuNPs/PEG/Dtx/FA) exhibits that the particles are spherical in shape with uniformly distributed over the substrate with an average dimension of 18 nm, without any aggregation (Fig. 5q–t). Figure 5d shows the SAED pattern of the AuNPs and the particles are highly crystalline in nature. The crystal lattice spacing distances of AuNPs is about 4.0786 Å. These results are in accordance with the (111), (200), (220) and (311) diffraction planes of gold (JCPDS ID # 00-002-1095). Thus, the results indicate that the gold particles are arranged in a face centered cubic crystals system with λ = 1.5406 Å. These results were compared with the XRD pattern. The SAED pattern of the gold nanoformulations was observed the particles are highly crystalline. Hence, this result confirms the step-by-step conjugation of Dtx encapsulated gold nanoformulations are highly crystalline nature without changing the crystalline morphology.

The elemental compositions of the synthesized gold nanoformulations were analyzed by EDS analysis (Fig. 5t). The results indicate the carbon of 34.09 wt%, nitrogen of 10.06 wt%, oxygen of 43.22 wt%, Sulfur of 0.22 wt% and gold of 12.40 wt% elements are present in the sample. These results suggest that the synthesized AuNPs nanoformulations are highly pure with good crystallinity. Hence, TEM and EDS analyses confirmed the microstructure, size, morphology and elemental composition of the AuNPs nanoformulations during the stepwise conjugation and drug formulation. The synthesized nanoformulations were highly stable in aqueous medium over the period of one year at room temperature.

### Field emission scanning electron microscope

**Figure 6:**
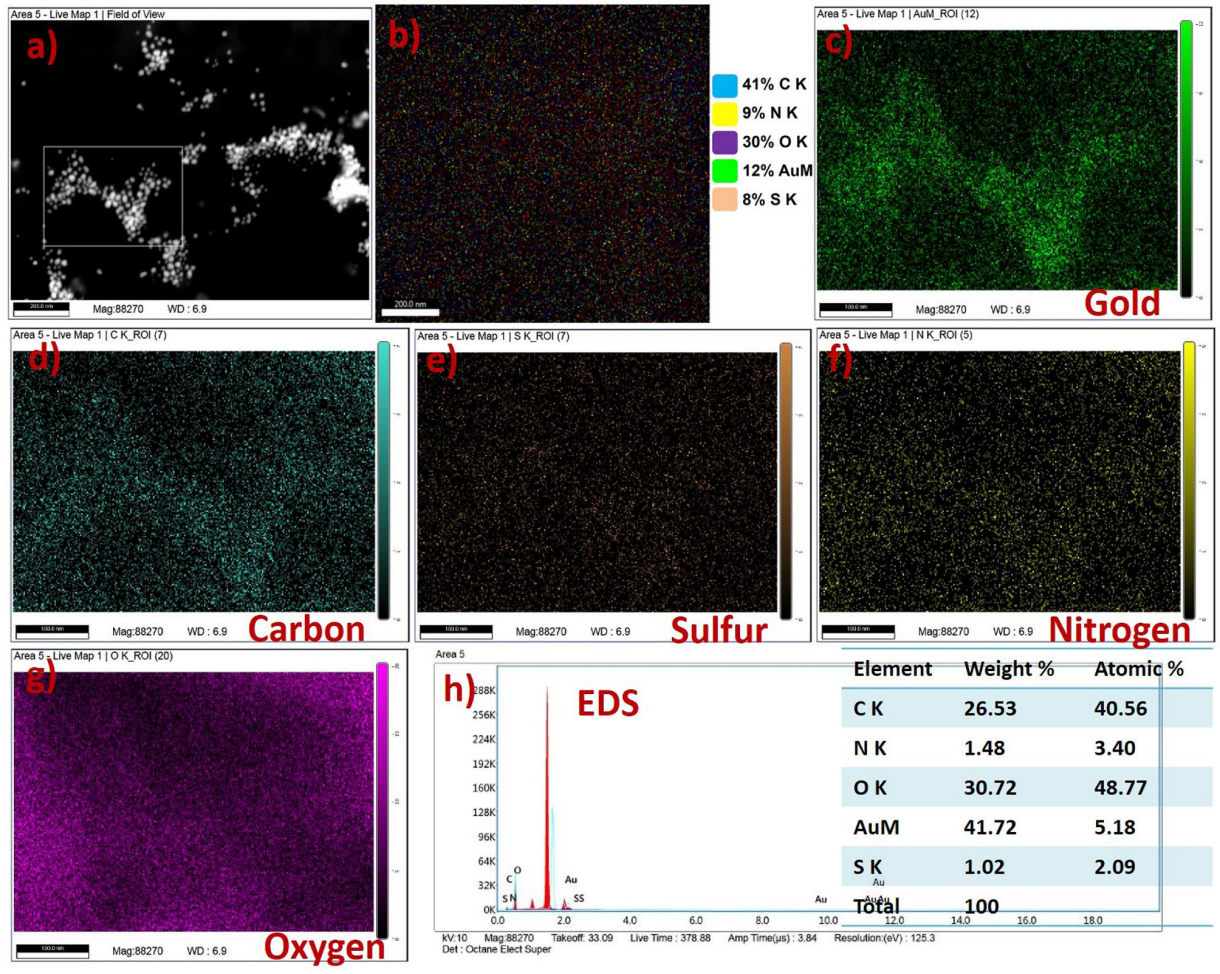
FESEM image and Elemental mapping with EDS spectrum of gold nanoformulations, (**a**) high magnification image gold nanoformulations with selected area for mapping analysis, (**b**) Elemental mapping images of gold nanoformulations with corresponding elements (C K = 41%, N K = 9%, O K = 30%, AuM = 12% and S K = 8%), (**c**) mapping image of metallic gold nanoparticles, (**d**) carbon, (**e**) sulfur, (**f**) nitrogen, (**g**) oxygen and (**h**) EDS spectrum of gold nanoformulations.

Elemental mapping analysis of the synthesized nanoformulations was evaluated by FESEM analysis. Figure 6a shows the AuNPs nanoformulations exhibited low and high magnification. The high magnification image of nanoformulations exhibited the particles are uniformly arranged over the substrate which is used for elemental mapping analysis. These images could be used as a selected area for the mapping analysis. Elemental mapping analysis of the nanoformulations was clearly indicating the Dtx and FA particles embedded with PEG functionalized AuNPs (Fig. 6b). The mapping images of nanoformulations showed the embedded elements of CK = 41%, NK = 9%, OK = 30%, metallic gold (AuM = 12%), and Sk = 8%. Figure 6c depicts the mapping image of gold particles in the metallic form which indicates the gold particles are present in the sample. The mapping images of carbon, sulfur, nitrogen, and oxygen were exhibited in Fig. 6d–g, respectively. This image represents the synthesized gold nanoformulations are highly pure without any impurities.

The elemental composition of the gold nanoformulations was evaluated by EDS spectroscopy as shown in Fig. 6h. The Dtx encapsulated FA conjugates PEG functionalized gold nanoformulations exhibited the elements of carbon (CK = 26.53%), sulfur (SK = 1.02), nitrogen (NK = 1.48%), oxygen (OK = 30.72), and gold (AuM = 41.72). This result represents the synthesized nanoformulations were systematically conjugated with AuNPs which exhibited the nanoformulations are highly pure. The detail FE-SEM images of AuNPs, PEG functionalized AuNPs, FA, AuNPs-PEG-FA, Dtx and gold nanoformulations were provided in supplementary section (Fig. S2a–i).

### XPS analysis

**Figure 7:**
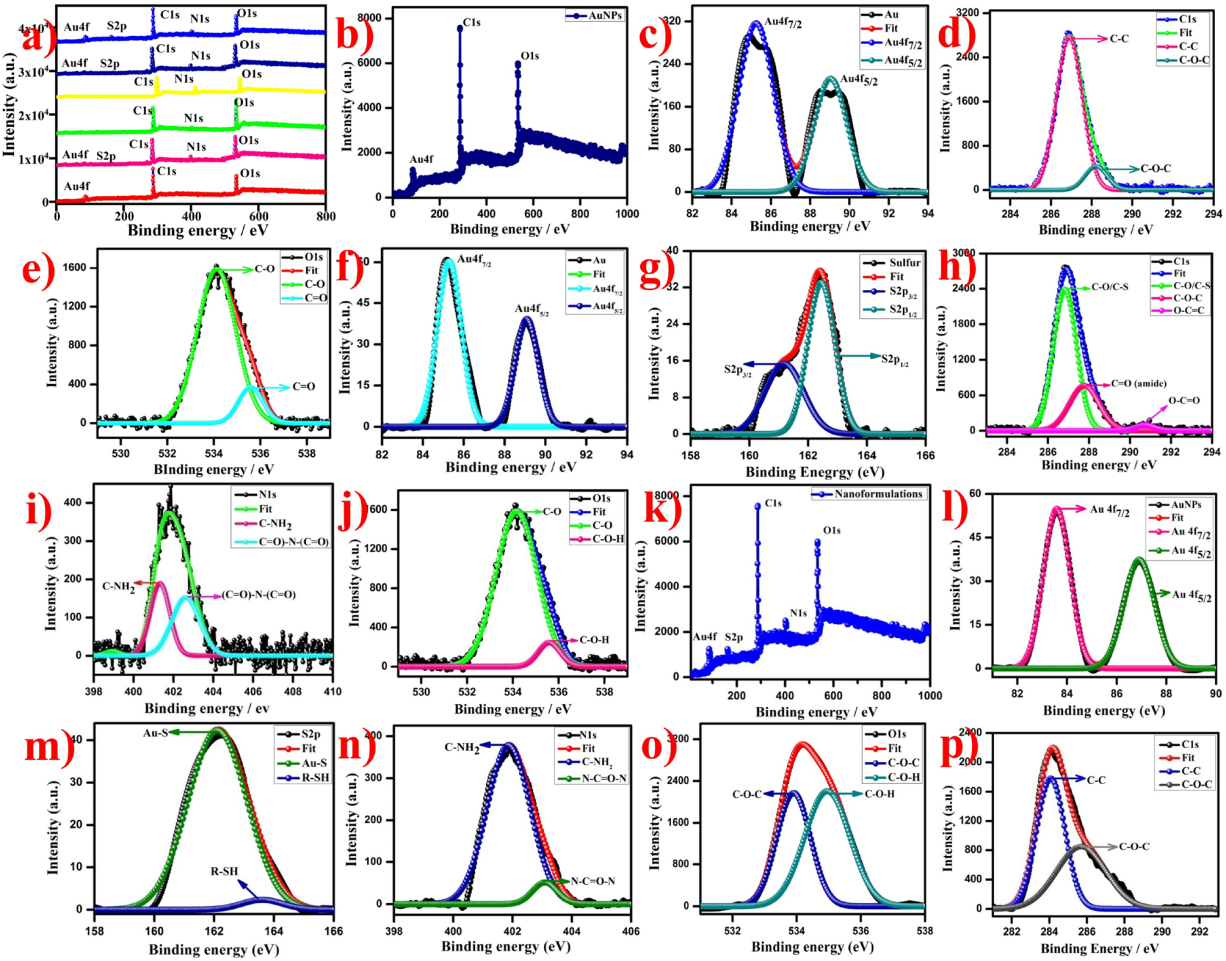
XPS spectra of gold nanoformulations, (**a**) overall survey spectra, (**b**–**d**) Survey spectrum of AuNPs and corresponding high resolution spectra as follows (**c**) Au4*f*_5/2_ and Au4*f*_7/2,_ (**d**) C1*s*, (**e**) O1*s*, (**f**–**j**) high resolution spectra of PEG functionalized AuNPs (**f**) Au4*f*, (**g**) S2*p*, (**h**) C1*s*, (**i**) N1*s* and (**j**) O1*s* and (**k**–**p**) Survey spectrum of gold based nanoformulations and corresponding high resolution spectra (**k**) survey spectrum, (**l**) Au4*f*, (**m**) S2*p*, (**n**) C1*s*, (**o**) N1*s* and (**p**) O1s.

The XPS analysis was used to determine the oxidation state and chemical composition of the nanoformulations. Figure 7a exhibits the survey spectrum of the AuNPs, AuNPs-PEG, FA, FA-AuNPs-PEG, Dtx, AuNPs-PEG-Dtx, and AuNPs-PEG-FA-Dtx, which observed the strongest band of gold, sulfur, carbon, nitrogen, and oxygen (Au4*f*, S2*p*, C1*s*, N1*s*, and O1*s*), respectively. Figure 7b depicts the survey spectrum of the AuNPs. The valence band and binding energy of 4*f* core level spectrum of AuNPs have observed the peaks at 85.3 eV and 88.9 eV corresponds to the Au 4*f*_7/2_ and Au 4*f*_5/2_ spin orbitals, respectively (Fig. 7c)^25^. These binding energies were compared to the respective core levels of bulk Au crystals. Evidently, this result exhibited the metallic types of gold (Au^0^) were present in the sample. Moreover, the resulted value of Au 4*f*_7/2_ and Au 4*f*_5/2_ exhibits the narrow width revealed that only a single element of gold was present in the system which indicates the synthesized AuNPs are highly stable. The Au4*f* core level spectrum of the SH-PEG-NH_2_ functionalized AuNPs exhibited the two components at 85.22 eV and 89.01 eV corresponds to the Au 4*f*_7/2_ and Au 4*f*_5/2_ of spin orbitals of PEG-AuNPs bonds with alkyl thiol self-assembled AuNPs (Au–S), respectively (Fig. 7f) whereas, the FA-PEG-AuNPs exhibited the two components at 83.55 eV and 86.88 eV corresponds to the core level of Au 4*f*_7/2_ and Au 4*f*_5/2_ spin-orbital, respectively and this value confirms the XPS traces of alkyl thiol self-assembled bonded over AuNPs (Fig. S3h). Figure S3a depicts the XPS survey spectrum of the free docetaxel which observed the traces of C1*s*, N1*s*, and O1*s*, whereas, Fig. 7k exhibits the XPS survey spectrum of gold nanoformulations. The Au4*f* core level XPS spectrum of the AuNPs-PEG-FA-Dtx exhibited the two components at 83.21 eV and 87.56 eV corresponds to the Au 4*f*_7/2_ and Au 4*f*_5/2_ spin-orbital, respectively (Fig. 7l). Hence, these XPS results confirm the functionalization of AuNPs nanoformulations.

The C1*s* core level spectra of the AuNPs exhibited the two XPS traces at 286.93 eV and 290.92 eV attributed to the C–O–H, C=C/C–O–C groups of the carbon atom, respectively (Fig. 7d), whereas, PEG functionalized AuNPs exhibited three XPS signals at 286.76 eV, 287.74 eV and 290.68 eV correspond to the C−O or C−S, C=O_(amide)_ and O–C=O group of the carbon atom, respectively (Fig. 7h)^70–72^. The appearance of the C1*s* XPS spectra of FA-AuNPs-PEG exhibited the signals at 284.02 eV, 285.69 eV ascribed to the C=C/C=N and C−C/C−H groups confirmed the attachment of FA, respectively (Fig. S3j). The Shifting of carbonyl carbon bond towards the lower binding energy level compared to PEG-AuNPs indicated the conversion of acid carbonyl group into amide carbonyl group and hence, the FA conjugation over the AuNPs-PEG via the formation of the amide bond. The C1*s* spectrum of FA exhibited the three XPS traces at 286.63 eV, 288.03 eV and 290.52 eV correspond to the C–N, C=O, and O–C=O, respectively (Fig. S3e), whereas, the Dtx spectrum exhibits the two signals at 297.96 eV and 301.02 eV corresponds to the O–C=O and C–O–C groups of carbon atoms (Fig. S3b). The Dtx encapsulated with FA conjugated PEG functionalized AuNPs exhibited the two components corresponding to the 284.05 eV and 286.69 eV corresponds to the C=C/C=N and C–C/C–H groups of the carbon atom, respectively (Fig. 7p).

The O1*s* XPS spectra of the AuNPs exhibited a band at 534.37 eV attributed to C–O/C=O groups of the oxygen atom (Fig. 7e)^25,58,73^. PEG functionalized AuNPs exhibited the two XPS traces at 534.19 eV and 535.53 eV corresponds to the C=O and C–O–H groups of the oxygen atom, respectively (Fig. 7j). The appearance of O1*s* XPS spectra of the AuNPs-PEG-FA exhibited the signals at 532.65 eV corresponds to the carbonyl group of FA and the minor component at 533.78 eV attributed to the C–O–C group of the oxygen atom (Fig. S3l). The O1s traces of FA exhibited the components at 533.83 eV and 534.18 eV corresponds to the C–O–C and C–O–H groups of the oxygen atom, respectively (Fig. S3g). The XPS spectra of Dtx exhibited the signals at 544.9 eV and 545.66 eV corresponds to the C-O and C=O groups of the oxygen atom present in the samples, respectively (Fig. S3d). The gold nanoformulations exhibit the O1*s* traces at 533.09 eV and 534.26 eV correspond to the free carboxylic acid and C–O–H group of the oxygen atom (Fig. 7o). This result showed that the O1*s* trace of the amide carbonyl groups of Dtx was encapsulated over AuNPs-PEG-FA via amide carbonyl groups at 533.09 eV, which confirms the step-by-step conjugation of Dtx encapsulated AuNPs-PEG-FA nanoformulations.

The S2*p* XPS spectra of the PEG functionalized AuNPs exhibited the two signals at 161.18 eV and 162.38 eV along with a 1.2 eV gap corresponds to the S2*p*_3/2_ and S2*p*_1/2_, (Fig. 7g)^74^ of the SH-PEG-NH2 functionalized AuNPs, respectively. Spampinato et al. reported that the unbounded sulfur observed at 164 eV corresponds to S2*p*_3/2_, while, the sulfur traces bound over the surface of AuNPs appeared at 162 eV. The appearance of the FA conjugated PEG functionalized AuNPs exhibited the S2*p*_3/2_ and S2*p*_1/2_ XPS signals at 162.02 eV, 163.56 eV and 164 eV correspond to the Au–S, R-SH and S groups of sulfur atoms, respectively (Fig. S3i). The gold nanoformulations exhibit the S2*p* traces at 162.18 eV and 163.80 eV corresponds to the Au–S and S groups of sulfur atoms present in the sample, respectively (Fig. 7m)^75^. Similarly, the entire spectrum exhibited the S2*p*_3/2_ XPS traces at 162 eV confirm the conjugation of PEG-FA-Dtx over AuNPs. The AuNPs did not show any N1s of XPS traces, whereas, N1*s* spectrum of the PEG-Functionalized AuNPs exhibited the XPS components at 401.27 eV and 402.58 eV corresponds to the C-NH2 and (C=O)N(C=O) groups of nitrogen atoms, respectively (Fig. 7i)^25,58,76^. The FA exhibited the N1s signals of 400.01 eV and 401.08 eV corresponds to the C–N/N−H and NH–C–O groups of nitrogen atoms, respectively (Fig. S3f), whereas, Dtx was observed the N1*s* energy of 401.2 eV and 402.78 eV corresponds to the C-NH2 and N–C=O–N groups of nitrogen atoms, respectively (Fig. S3c). The appearance of the FA conjugated PEG functionalized AuNPs exhibited the N1*s* XPS signals at 399.93 eV and 400.67 eV corresponds to the C–N/N−H and NH–C–O groups of the nitrogen atoms, respectively (Fig. S3k). The gold nanoformulations exhibited the N1*s* XPS components at 401.07 eV and 403 eV corresponds to the C–NH2 and N–C=O–N groups of the nitrogen atom present in the nanoformulations, respectively (Fig. 7. (n)). Hence, these XPS spectra support the stepwise formation of Dtx encapsulated FA conjugated PEG functionalized AuNPs. The synthesized gold nanoformulations are highly pure without any impurity peaks were appeared.

### Raman spectra of gold nanoformulations

**Figure 8:**
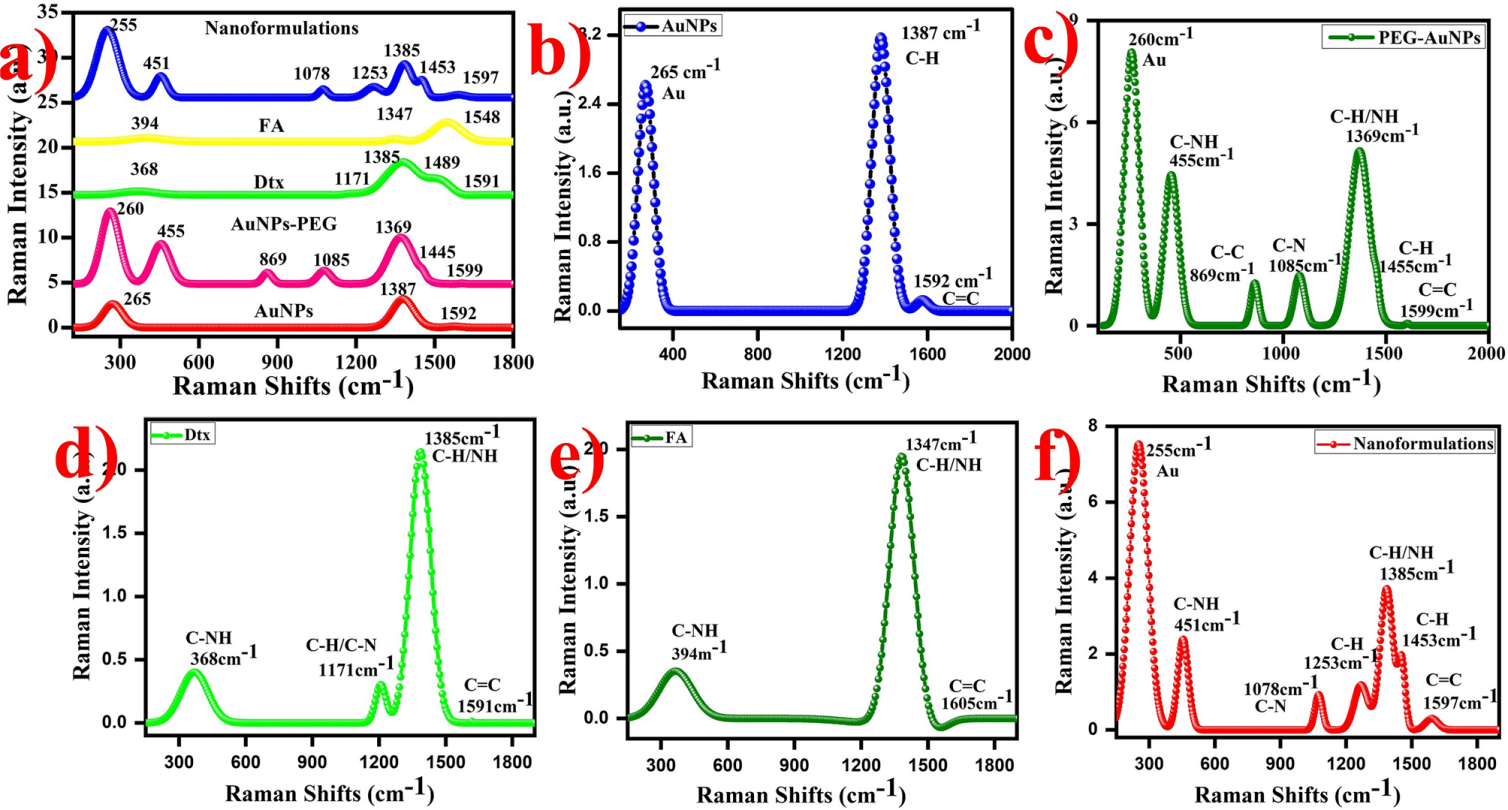
Raman spectra of the gold nanoformulations (**a**) Combined spectra of AuNPs nanoformulations, (**b**) AuNPs (**c**) AuNPs-PEG, (**d**) Dtx, (**e**) FA and (**f**) AuNPs-PEG-FA-Dtx.

Raman spectroscopy is used to identify the structural fingerprints of the particles. Raman spectra of AuNPs exhibited band at 267 cm^−1^ corresponds to the Au^(III)^ to Au^0^. The shoulder band at 1385 cm^−1^ and 1585 cm^−1^ is corresponds to the C–H and C=C of carbon (citrate ions), respectively (Fig. 8b)^64^, whereas, PEG functionalized AuNPs exhibited the Raman spectra appeared the signals at 260.81 cm^-1^,455 cm^−1^ (C–NH amide), 869 cm^−1^ (C–N in-plane bending), 1085 cm^−1^(C–C stretching of carbonyl groups), 1369 cm^-1^, 1445 cm^−1^ and 1599 cm^−1^ corresponds to the Au^0^, C–O, C–H and C–O–H strongly observed on the surface, respectively (Fig. 8c). The appearance of the FA exhibited the bands at 394 cm^−1^, 1347 cm^−1^ and 1548 cm^−1^ corresponds to the amide group, carbonyl and carboxylic acid groups were present in the sample, respectively (Fig. 8d)^65^. The Dtx exhibited the Raman spectra observed the band at 368 cm^−1^, 1171 cm^−1^, 1385 cm^−1^, 1489 cm^−1^ and 1591 cm^−1^ corresponds to the amide group (C–NH), carbonyl (C–H) groups, C–H, N–H and C=C present in the Dtx surfaces, respectively (Fig. 8e)^77^.

The Raman spectra of the synthesized gold nanoformulations exhibited the band at 255 cm^−1^, 451 cm^−1^, 1078 cm^−1^, 1253 cm^−1^, 1385 cm^−1^, 1453 cm^−1^ and 1597 cm^−1^ (Fig. 8f). From the spectra, the shoulder peak at 260 cm^−1^ corresponds to the Au^0^ strongly observed in the surface. The peaks at 451 cm^−1^, 1078 cm^−1^ and 1253 cm^−1^ are attributed to the C–NH, C–N, C–H of carbonyl and amine groups are present in the sample, respectively. These peaks indicates the conjugation of AuNPs with FA and Dtx. The major peaks at 1385 cm^−1^, 1455 cm^−1^ and 1597 cm^−1^ are ascribed to the C–H/NH, C–H and C=C of bending frequency of amine and carbonyl groups due to the formation of FA and Dtx. The combined Raman spectra of gold nanoformulations are provided due to comparison between the nanoparticles (Fig. 8a). Thus, the results suggest that the synthesized AuNPs are highly pure.

### In vitro analysis of gold nanoformulations

The encapsulation efficiency of gold nanoformulations was observed at approximately ~ 95%. Here, 1 mM con of Dtx solution was prepared and encapsulated with PEG functionalized FA conjugated AuNPs by the non-covalent method. The resulted nanoformulations were sonicated and centrifuged at 10,000 rpm at 0 °C for 15 min. The obtained solution was used for further characterization and drug release studies.

### In vitro drug release studies

**Figure 9:**
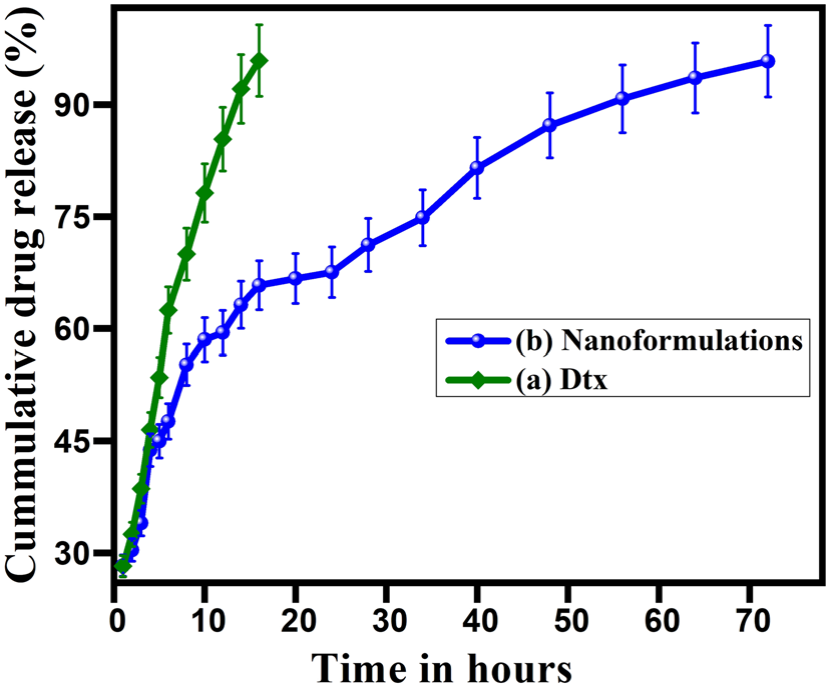
In vitro drug release profile of (**a**) docetaxel and (**b**) gold nanoformulations in PBS solution at pH 7.4.

The controlled drug discharge profile exhibited the drug was smoothly circulated in blood over the period and which minimizes side effects, reduces drug usages, and maximizes therapeutic efficacy. In vitro, drug release pattern of free docetaxel and Dtx encapsulated gold nanoformulations were evaluated using the dialysis membrane diffusion method. The release pattern was expressed by the percentage of cumulative drug release against time in hours as shown in Fig. 9. Initially, a very fast and continuous drug (Dtx) release behavior was observed (i) burst discharge up to 45 to 50% in 1.5 h followed by (ii) the sustained discharge at the maximum level of 96% in 18 h (Fig. 9a). The cumulative drug discharge behavior of Dtx encapsulated gold nanoformulations were observed during the prolonged discharge. The Dtx encapsulated AuNPs nanoformulations exhibited the tri-phasic release pattern was observed. At first, burst discharge was observed about 28% of Dtx released from the nanoformulations in 16 h than, sustained discharge up to 35 h and finally slow with constant discharge up to 72 h at the maximum of the release of 96%. These results clearly indicate that the maximum Dtx release in gold nanoformulations was observed up to 96% and the discharge behavior (rate and style) varies which exhibited in Fig. 9b. This drug release system contains a tri-phasic discharge system, initial burst release due to the release of drugs from the surface of nanoparticles followed by the second phase which shows a sustained release due to the release of drugs from the matrix which represents the Fickian diffusion and finally, slow with a constant discharge of drugs from the nanoformulations. This release pattern has followed the characteristics of Higuchi’s square root kinetics with a correlation coefficient of r = 0.9996. This drug discharge as follows the diffusion with erosion mechanism.

### Cytotoxicity analysis of gold nanoformulations

**Figure 10:**
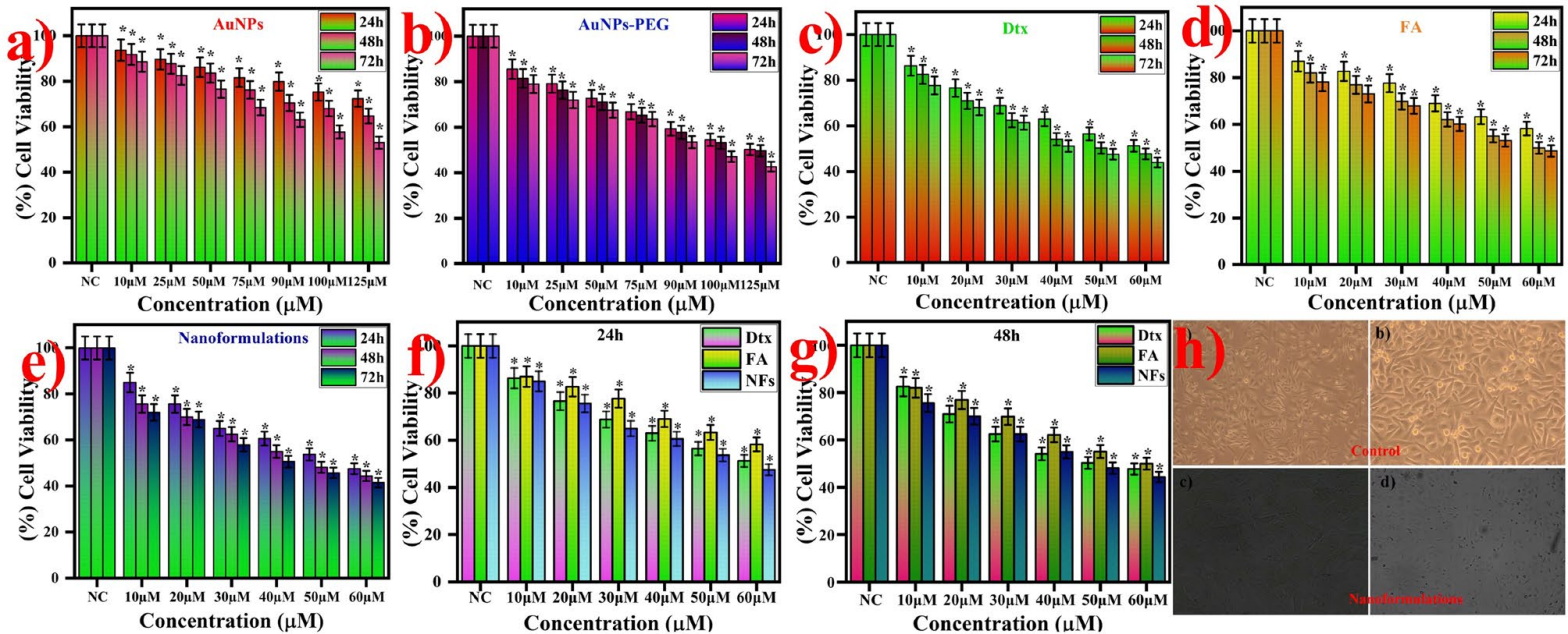
In vitro cytotoxicity analysis of gold nanoformulations against PC3 cell lines at different time intervals such as 24 h, 48 h, and 72 h using premix WST cell proliferation assay kit. (**a**) AuNPs, (**b**) PEG-AuNPs, (**c**) Dtx, (**d**) FA (**e**) AuNPs nanoformulations and (**f**,**g**) Comparison between Dtx, FA and nanoformulations at 24 and 48 h post treatment, and (**h**) PC3 cell images at control and nanoformulations. [*Corresponds to the statistical significance values (p < 0.05)].

The anticancer activity of the synthesized gold nanoformulations includes AuNPs, AuNPs-PEG, FA, Dtx and Dtx encapsulated gold nanoformulations (AuNPs-PEG-FA-Dtx) were investigated against prostate cancer cell of PC3 cells (Fig. 10a–h). Figure 10a and b clearly indicates that the cell viability of the AuNPs and PEG-AuNPs was observed the 75% and 60%, respectively at 72 h post treatment, this difference due to the amine and thiol groups are attached over the AuNPs surface. The above 60% of cell viability was detected at 72 h treatment of FA (Fig. 10d). This result indicates that the AuNPs-PEG-FA showed less cytotoxicity effect on PC3 cells up to 60 μM of AuNPs and 50 μM of FA over 72 h. The anticancer drug of docetaxel was detected the 50% cell viability of 40 μM (32.28 μg/mL) at 72 h treatment. IC_50_ was determined to be 40 µM for free docetaxel at 48 h (Fig. 10c). At the same time point the concentrations for AuNPs, AuNPs-PEG and FA were also determined at which we have maximum cell death, thus contributing to an optimum value for the formation of nanoformulations. We have determined that at the maximum concentration of 60 µM for AuNPs and the cell death has approximately 25% of the control, while FA has almost 80% cell viability in the 50 µM concentrations. The results provide an idea of the optimal concentrations of the components of the nanoformulation with better cytotoxic activity.

Interestingly, AuNPs-PEG-FA-Dtx exhibited only 60–70% of cell viability at the concentration of 10 µM to 60 µM at 24 h treatment, and the PC3 cells proliferation was decreased about 75%, 68%, 56%, 47 and 39% over 73 h (Fig. 10e). The result indicates that the potential cytotoxic effect of AuNPs-PEG-FA-Dtx against PC3 cells at 10 µM to 60 µM. Moreover, this results compared with Dtx to an increase in the cytotoxicity up to 50% and the significance (p < 0.05) results also investigated. The comparison between Dtx, FA and nanoformulations were observed different cytotoxic activity against prostate cancer cell lines as shown in Fig. 10f, g. The conjugation of FA into AuNPs-PEG-Dtx could increase the targeting efficacy of the obtained nanoformulations due to the relatively higher occurrence of folate receptors present on the surface of the PC3 cancer cells. However, the anticancer activity of the gold nanoformulations possesses to their anti-folate activity of folate-Dtx released from the AuNPs-PEG-FA-Dtx under lysosomal pH condition in PC3 cells. Hence, the anticancer activity of the AuNPs-PEG-FA-Dtx possesses potential nanoformulations for the promising chemotherapeutic agents for the treatment of various cancers. The morphological analysis of AuNPs nanoformulations, based on the cytotoxicity effect of the PC3 cell lines treated against nanoformulations has been visualized by optical microscope. The corresponding untreated cells as well as cells treated images are shown in Fig. 10. Figure 10h depicts the normal cells and Dtx encapsulated nanoformulations treated cells with various spots of the 96 well plates. This result suggests that the cytotoxicity results are in accordance with the MTT assays.

## Conclusion

In this work, we have established a simple, stable and highly promising AuNPs nanoformulations for targeted drug delivery to prostate cancer. The SH-PEG-NH_2_ was successfully functionalized over citrate capped AuNPs without any agglomeration at pH 6.5 and FA conjugated onto AuNPs-PEG by EDC/NHS coupling and covalent linkage method. The anticancer drug, Dtx was encapsulated within AuNPs-PEG-FA by the non-covalent linkage method. The FTIR, Raman and XPS structural analysis confirmed the step-by-step chemical bonding of FA over AuNPs-PEG and Dtx over AuNPs-PEG-FA nanoformulations. The XRD and SAED pattern confirms the AuNPs nanoformulations exhibited the face centered cubic crystal structure of AuNPs. The HR-TEM and FE-SEM confirm the morphology and microstructure of the gold nanoformulations are spherical in shape with an average size of 16 nm and 18 nm for AuNPs and nanoformulations, respectively. The elemental mapping analysis also evident that the encapsulation of Dtx over AuNPs and the encapsulation efficiency of the Dtx was found to be ~ 96%. The drug release profile of the nanoformulations was found three different kinetics of slow, burst and sustained release which mainly follows the Higuchi kinetics. The cell viability of the gold nanoformulations treated against PC3 cells increases with increasing time, indicating that the nanoformulations decelerate cell proliferation of PC3 cells. Hence, the synthesized AuNPs-PEG-FA-Dtx nanoformulations could be used as an effective and alternative drug delivery system for prostate cancer treatment to achieve improved therapeutic efficacy with decreased drug dosage.

## Supplementary Information

Supplementary Information.
