# Supplementary material for: An effective strategy for development of docetaxel encapsulated gold nanoformulations for treatment of prostate cancer
Source: Sci Rep. 2021 Feb 2;11:2808. doi: 10.1038/s41598-020-80529-1 (PMC7854673; doi:10.1038/s41598-020-80529-1)
Supplement: Supplementary file 1 — Supplementary Information. [file 41598_2020_80529_MOESM1_ESM.doc]

An Effective Strategy for development of Docetaxel encapsulated Gold Nanoformulations for Treatment of Prostate Cancer

S. Thambiraj^1^, R. Vijayalakshmi^2^ and D. Ravi Shankaran^1^,*

Supplementary Results


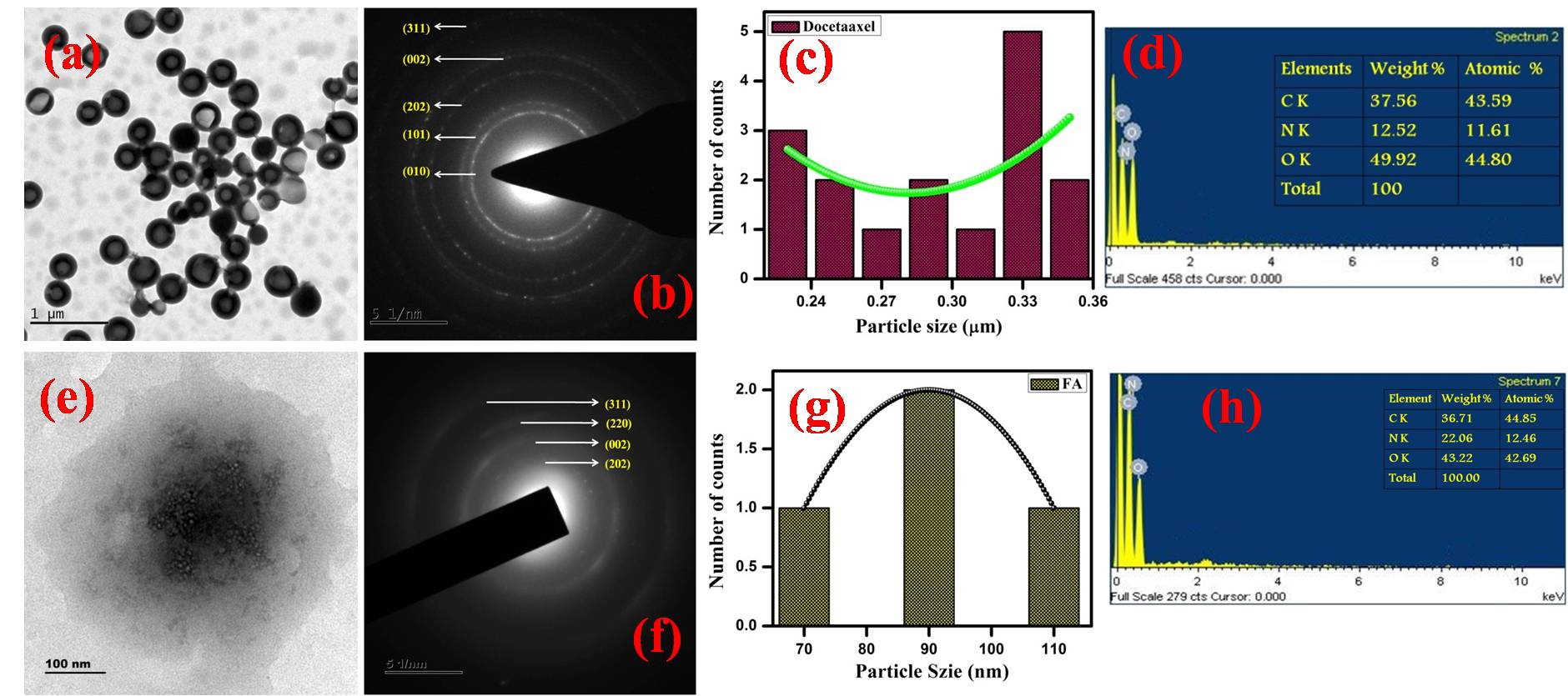


Fig.S1. TEM image of (a-d) Dtx TEM image, SAED, particle size distribution and EDS spectrum followed by the same characteristics carried out for FA (e-h).

**FE-SEM images of gold nanoformulations**

The surface morphology and elemental mapping with EDS of the synthesized gold based nanoformulations were evaluated by FE-SEM analysis as shown in fig.S2. Fig.S2. (a) depicts the FE-SEM micrograph of AuNPs which exhibited the particles are spherical in shape with uniformly distributed in the aluminum substrate. The average size of the AuNPs was found to be 22 nm. Fig.S2. (b) shows the PEG functionalized AuNPs was observed at 200 nm magnification and the particles are spherical in shape with an average diameter of 28 nm. Fig.S2.(c) shows the low magnification image of docetaxel and the particles are randomly dispersed over the aluminum substrate. These particles are spherical in shape with core-shell-like morphology was observed. The average size of prepared Dtx was found to be 28 nm. Fig.S2.(d) depicts the docetaxel encapsulated AuNPs at 6µm magnification and the particles are spherical in shape and AuNPs were encapsulate with Dtx. Fig.S2.(e) indicates the low magnification (2µm) images of folic acid which indicates the particles are spherical in shape with the diameter of 440 nm. Fig. S2.(f) shows the folic acid conjugated AuNPs at 500nm magnification and AuNPs were conjugated with FA. Fig.S2.(g) shows FE-SEM image of AuNPs/PEG/Dtx/FA nanoformulations and the particles are spherical in shape with a smooth surface, without any aggregation. Fig.S2.(h and i) shows the EDS spectra of the AuNPs and AuNPs nanoformulations.


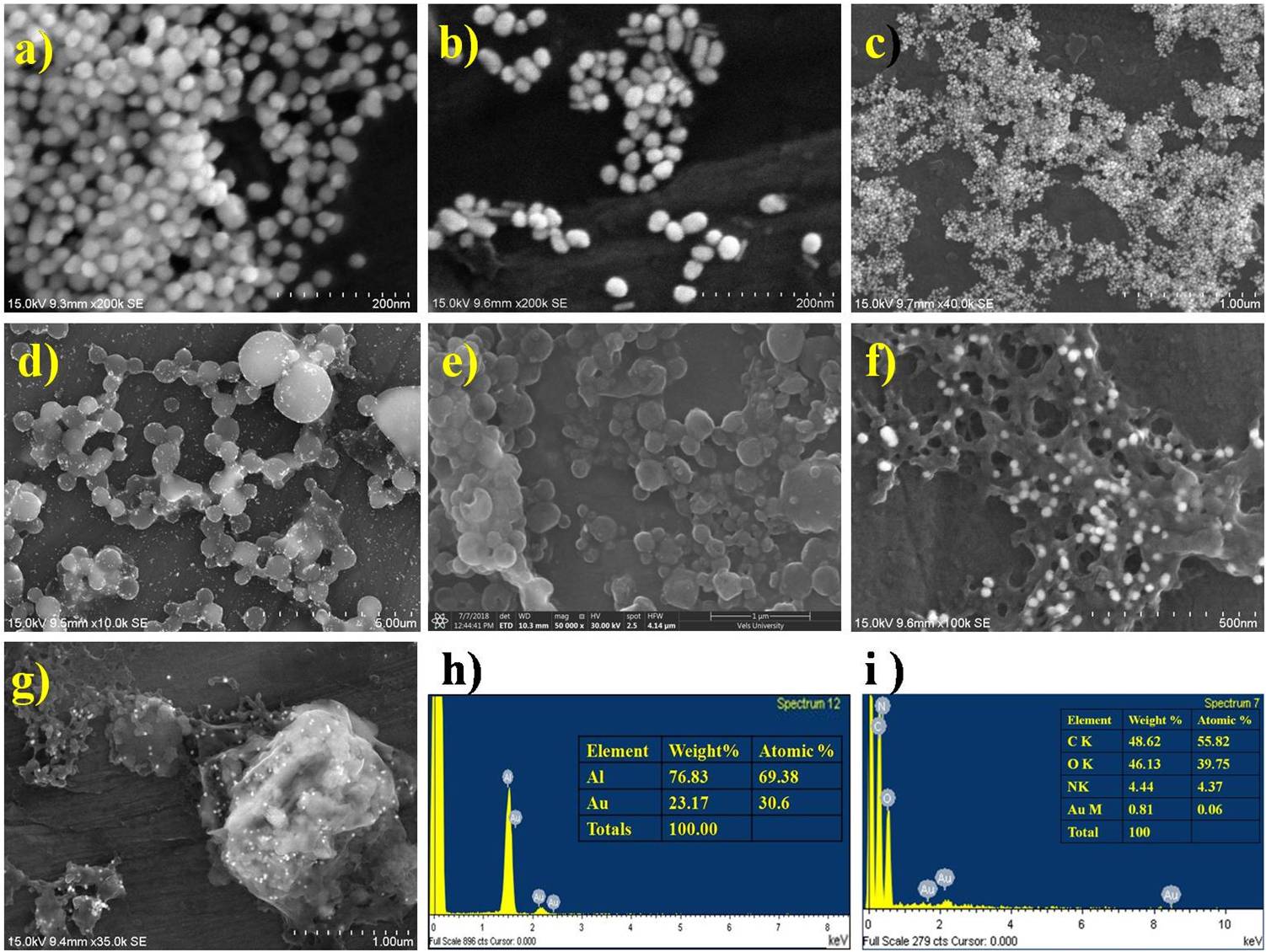


Fig.S2. FE-SEM images of gold based nanoformulations (a) Spherical shaped AuNPs, (b) PEG functionalized AuNPs, (c) anticancer drug of Dtx, (d) Dtx loaded AuNPs, (e) FA, (f) FA loaded AuNPs, (g) gold based nanoformulations (AuNPs/PEG/Dtx/FA) (h) EDS spectrum of AuNPs, and (i) EDS spectrum of gold based nanoformulations.


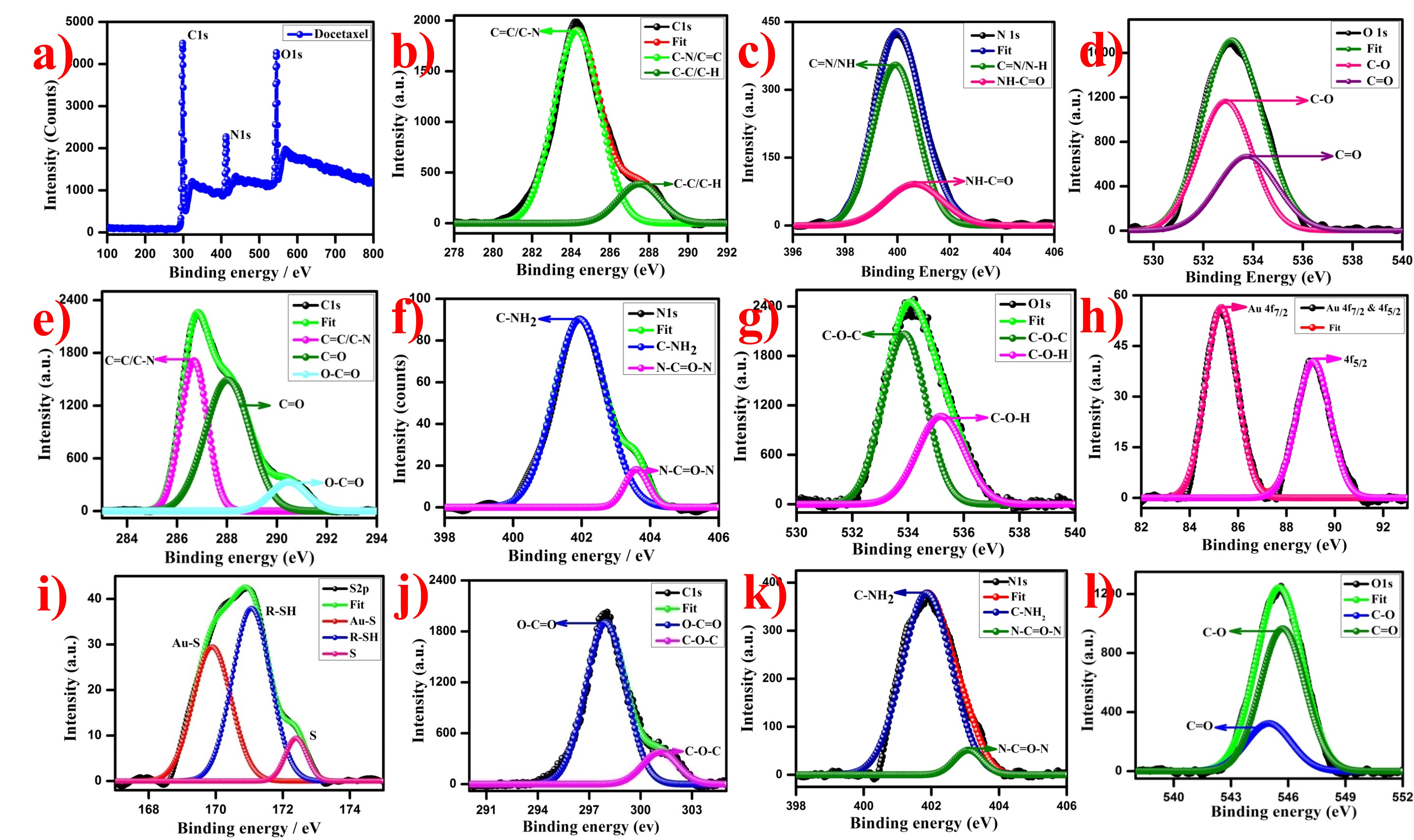
Fig.S3.XPS spectra of (a-d) survey spectrum corresponding high resolution spectrum of docetaxel, (e-g) high resolution spectra of FA corresponding core levels of C1s, N1s and O1s and (h-l) high resolution image of FA conjugated AuNPs-PEG with high resolution spectra Au4f, S2p, C1s, N1s, and O1s, respectively.
